# A Diabetes Pay-for-Performance Program and Risks of Cancer Incidence and Death in Patients With Type 2 Diabetes in Taiwan

**DOI:** 10.5888/pcd14.170012

**Published:** 2017-10-05

**Authors:** Hui-Min Hsieh, Jiun-Shiuan He, Shyi-Jang Shin, Herng-Chia Chiu, Charles Tzu-Chi Lee

**Affiliations:** 1 Department of Public Health, Kaohsiung Medical University, Kaohsiung, Taiwan; 2 Department of Medical Research, Kaohsiung Medical University Hospital, Kaohsiung, Taiwan; 3 Department of Internal Medicine, Kaohsiung Municipal Ta-Tung Hospital, Kaohsiung Medical University, Kaohsiung, Taiwan; 4 Graduate Institute of Medical Genetics, Kaohsiung Medical University, Kaohsiung, Taiwan; 5 Division of Endocrinology and Metabolism, Kaohsiung Medical University, Kaohsiung, Taiwan; 6 Department of Healthcare Administration and Medical Informatics, Kaohsiung Medical University, Kaohsiung, Taiwan; 7 Research Education and Epidemiology Center, Changhua Christian Hospital, Changhua, Taiwan; 8 Department of Health Promotion and Health Education, National Taiwan Normal University, Taipei, Taiwan

## Abstract

**Introduction:**

We sought to evaluate the effects of diabetes disease management through a diabetes pay-for-performance (P4P) program in Taiwan on risks of incident cancer and mortality among patients with type 2 diabetes.

**Methods:**

We conducted a longitudinal observational cohort study using 3 population-based databases in Taiwan. Using propensity score matching, we compared patients with type 2 diabetes who enrolled in a P4P program with a similar group of patients who did not enroll in the in P4P program (non-P4P). Primary end points of interest were risks of incident cancer and all-cause, cancer-specific, and diabetes-related mortality. Total person-years and incidence and mortality rates per 1,000 person-years were calculated. Multivariable Cox proportional hazard models and competing risk regression were used in the analysis.

**Results:**

Overall, our findings indicated that the diabetes P4P program was not significantly associated with lower risks of cancer incidence, but it was associated with lower risks of all-cause mortality (adjusted subdistribution hazard ratio [aSHR], 0.59; 95% confidence interval [CI], 0.55–0.63), cancer-specific mortality (aSHR, 0.85; 95% CI, 0.73–1.00), and diabetes-related mortality (aSHR, 0.54: 95% CI, 0.49–0.60). Metformin, thiazolidinediones, and α glucosidase inhibitors were associated with lower risks of cancer incidence and cancer-specific mortality.

**Conclusion:**

Our findings provide evidence of the potential benefit of diabetes P4P programs in reducing risks of all-cause mortality and competing causes of death attributable to cancer-specific and diabetes-related mortality among type 2 diabetes patients.

## Introduction

Diabetes mellitus and cancer are common, serious global health problems that contribute substantially to health care costs. A 2014 report from the International Diabetes Federation estimated that more than 387 million people worldwide have diabetes, and by 2035 this number will rise to 592 million; 4.9 million deaths and at least US $612 billion in health expenditure resulted from diabetes in 2014 (1). Diabetes is considered a strong independent predictor of vascular diseases (2). Growing evidence suggests a possible association between diabetes (especially type 2 diabetes) and site-specific cancer risks (eg, liver, breast, colorectal), as well as cancer mortality (3,4).

Although the causal mechanisms for the association between diabetes and cancer are not clear, potential risk factors common to both are recognized, including demographic (age, sex, race/ethnicity), genetic, and lifestyle-related (obesity, diet, physical activity, tobacco or alcohol consumption) risk factors. Potential mechanisms for a possible biologic link between diabetes and cancer include insulin resistance, hyperinsulinemia, hyperglycemia, and chronic inflammation (5,6). Most empirical studies focus on examining the intervention effect of glucose-lowering medication therapies (metformin, thiazolidinediones, sulfonylureas) on cancer risks or cancer prognosis, which in turn may influence cancer-specific mortality. However, the results regarding associations with cancer risk are mixed (3–5). Additional studies examined primarily the association between single healthy lifestyle choices (weight control, healthy diet, physical activity) and the risks of certain types of cancer (5,7). To the best of our knowledge, few studies have investigated the extent to which integrated interventions through a comprehensive and multidisciplinary diabetes management program might mitigate cancer risks and cancer mortality.

Pay-for-performance (P4P) or value-based purchasing programs have been embraced by many developed nations as a strategic tool to stimulate delivery of long-term, multidisciplinary diabetes management and to allow investment of less money on incentives while efficiently improving diabetes care quality (8–10). For example, the United Kingdom’s Quality and Outcome Framework and Australia’s P4P program pay bonuses to reward improvements in care for diabetes patients (9,11). In Taiwan, a diabetes P4P program was implemented nationwide by Taiwan’s National Health Insurance Administration (NHIA) at the end of 2001 to provide comprehensive diabetes management by following the American Diabetes Association’s clinical practice guidelines (12). Comprehensive care through diabetes P4P programs may enhance quality of care and prevent or delay vascular complications (12,13) or reduce risks of all-cause mortality in patients with diabetes (13). However, evidence of whether comprehensive diabetes care through a P4P program has any effect on incidence of types of cancer, or competing risks for cancer-specific or diabetes-related death, is limited.

This study aimed to examine the effects of comprehensive diabetes care provided through a nationwide diabetes P4P program in Taiwan on risks of cancer incidence and mortality among patients with type 2 diabetes. We hypothesized that modifying lifestyle-related risk factors with a comprehensive diabetes P4P program or administration of glucose-lowering medication therapies may prevent or delay incident cancer. We conducted an observational intervention and comparison cohort study using data from 3 longitudinal population-based databases in Taiwan to examine the extent to which the P4P program and other risk factors were associated with cancer incidence and competing causes of death (cancer-specific and diabetes-related) in patients with type 2 diabetes who enrolled in the P4P program compared with a group of diabetes patients who did not participate.

## Methods

A diabetes P4P program was implemented by Taiwan’s NHIA in 2001 to improve the quality of health care for diabetes patients. The program consists of several features (12). First, patients with diabetes who have at least 2 outpatient visits within 3 months in the same health care institution are eligible to enroll in the P4P program. Second, only physicians who specialize in metabolic disorders or endocrinology or who attend a training program for diabetes care are eligible to participate in and voluntarily enroll patients into this P4P program. Third, medical care teams are expected to work as coordinated physician-led multidisciplinary teams adhering to the American Diabetes Association’s clinical guidelines. Fourth, in addition to regular and usual care, P4P patients receive extra comprehensive care, including medical history assessment, physical examination, laboratory evaluation, management plan evaluation, self-management and health education. Finally, participating P4P physicians receive extra incentive payments in addition to regular physician fees depending on incentive targets for improving process (eg, documented hemoglobin A1c [HbA1c] or low-density lipoprotein tests) and intermediate outcomes (eg, higher percentages of patients with controlled HbA1c or low-density lipoprotein) (12,14).

### Study design and data source

We conducted a longitudinal observational intervention and comparison cohort study using 3 population-based databases in Taiwan. One database was the 2-million-sample longitudinal health and welfare database from 2000 through 2010, a nationally representative random sample of NHIA beneficiaries in 2005, from which we obtained information on patient comorbid conditions, health provider characteristics, and billing variables to identify patients enrolled in the P4P program. The second was a database containing death registry data from 2000 through 2010, which provides accurate death dates and cause-of-death information. The third was a national cancer registry, from which we obtained accurate cancer diagnosis data from 1979 through 2010. The 3 databases were linked with encrypted patient identifiers, and all data analysis was completed in the Health and Welfare Data Science Center, the Ministry of Health and Welfare in Taiwan in 2015 and 2016. This study was approved by the institutional review board at Kaohsiung Medical University Hospital.

### Study population

Diabetes patients were identified by a primary diabetes diagnosis code (*International Classification of Diseases, Ninth Revision, Clinical Modification* [ICD-9-CM], code 250) on at least 2 outpatient claims or at least 1 inpatient claim in each year during the patient identification period from 2003 through 2005. We first excluded patients with type 1 diabetes (ICD-9-CM codes 250.x1 or 250.x3), because they made up less than 3% of diabetes patients, and patients aged younger than 18 years on the date of first diabetes diagnosis during the patient identification period. Among eligible diabetes patients, we then identified newly enrolled P4P patients with at least 2 billing codes with “P14x” (internal code) for different office visits in the claims during the patient identification period and defined the date of first enrollment as the index date. We identified non-P4P diabetes patients for the comparison groups who were not found to be enrolled in the P4P program during the patient identification period or the follow-up period. Given that non-P4P patients lacked P4P enrollment index dates, we randomly assigned index dates based on the dynamic frequency distribution of time exposure to the P4P intervention from the P4P group (15). Using the cancer registry database, we excluded patients with any cancer diagnosis from the first year of the cancer registry in 1979 to the index date. Totals of 9,450 P4P and 32,923 non-P4P patients with diabetes were identified.

Propensity score matching (PSM) was used to determine adequate comparison groups to avoid potential confounding by selection bias and confounding factors. We created propensity scores that predicted the probability of enrollment into the diabetes P4P program in a logistic regression model. The PSM caliper matching method with 1-to-1 match was used to match intervention group with comparison group members based on propensity score (16,17). This approach finds the nearest distance of probabilities for the estimated propensity score to determine the best matches with the smallest standard deviations between intervention and comparison groups. If more than 1 potential comparison group member has the same propensity score as an intervention group member, the algorithm randomly selects one for inclusion in the comparison group (16,17). We also calculated standardized differences of covariates between phases, and all differences less than 10% indicated acceptable matching (18). Covariates included demographic characteristics (age, sex, highest level of education, urban or rural residence), baseline comorbid conditions (eg, the diabetes complications severity index [DCSI] [19] and chronic illness with complexity index [CIC] [20]), baseline antidiabetes drug use from 1 year before to the index date, and the most frequent health institution characteristics (accreditation level and geographic location). After PSM matching, 9,329 P4P and non-P4P matched pairs were studied.

### Variable definitions

The major end points were risks of cancer incidence and all-cause, cancer-specific, and diabetes-related mortality, with outcomes for P4P and non-P4P patients being compared. Overall cancer and common types of cancer (eg, breast, colorectal, liver) among diabetes patients were identified using ICD-9-CM diagnosis codes in the national cancer registry data. Cancer-specific and diabetes-related mortality were identified from causes of death based on the ICD-9 and ICD-10 diagnosis codes in the national death registry data. We defined diabetes-related death given the major risks of diabetes complications and prognosis that may cause death, including diabetes mellitus, macrovascular complications (eg, cardiovascular, cerebrovascular, and peripheral vascular diseases) and microvascular complications (eg, nephropathy and neuropathy diseases) (19). To compare groups, we followed each P4P and non-P4P patient from the index date to the first event date, death date, or study end date on December 31, 2010, whichever came first. We then calculated and compared total person-years for each patient and incidence and mortality per 1,000 person-years between P4P and non-P4P patients with diabetes given different end points.

Several baseline characteristics that may affect outcomes were included as control variables. Patient demographic covariates included age, sex, highest level of education, and urban or rural residence. The DCSI and CIC within 1 year before the index date were used to measure baseline comorbidity. These indexes are frequently used in studies (19,20). The DCSI has 7 categories of complications by ICD-9-CM code: cardiovascular complications, nephropathy, retinopathy, peripheral vascular disease, stroke, neuropathy, and metabolic disorders. The CIC index includes nondiabetes physical illness complexity, diabetes-related complexity, and mental illness or substance abuse complexity. We excluded cancer and diabetes-related complexity when constructing the CIC index to avoid the duplication of the comorbidity identified by the DCSI and cancer disease (21). Additionally, we controlled for frequently prescribed antidiabetes medications used during the follow-up period on the basis of the Anatomical Therapeutic Chemical Classification System (ATC codes), measured as 0 or 1 binary variables for identifying whether metformin (ATC code A10BA), sulfonylureas (SUs, ATC code A10BB), thiazolidinediones (TZDs, ATC code A10BG), or α glucosidase inhibitors (AGIs, ATC code A10BF) were prescribed.

Because the P4P program requires health staff to work as a team and cost structures may differ by health institution, the characteristics of the most frequently seen health care provider were included to identify the resources and capacities of individual health care institutions; characteristics included accreditation levels (medical center, regional hospital, local hospital, or clinic) and geographic locations (Taipei, northern area, central area, southern area, Kao-Ping, or eastern area).

### Statistical analysis

In addition to the PSM approach, we used multivariable Cox proportional hazard models to estimate the likelihood of all-cause mortality comparing matched P4P and non-P4P patients. We tested the proportional hazard assumption based on scaled Schoenfeld residuals after fitting Cox proportional hazard models and found no evidence that the proportional-hazard assumption was violated (22). To account for the competing causes of death, cancer-specific death, and diabetes-related death and for the competing risks of cancer incidence and death, we created competing risk regression models; we calculated subdistribution hazard ratios (SHRs) and 95% confidence intervals (CIs) (23). Potential confounding variables (Table 1) were controlled for. Patients were clustered within matched pairs and health care institutions to control for unequal error variances across matched pairs and health care providers. All statistical operations were performed by using SAS version 9.4 (SAS Institute, Inc) and Stata version SE 13 (StataCorp LP). A *P* value of less than .05 was considered significant.

**Table 1:** Patient and Health Care Institution Characteristics Among P4P and Non-P4P Patients With Type 2 Diabetes, Taiwan

Variable	Before PSM Matching			After PSM Matching			
	P4P	Non-P4P	*P* Value	P4P	Non-P4P	*P* Value	Standardized Difference, %^a^
**No.**	9,450	32,923	NA	9,329	9,329	NA	
**Patient Characteristics**							
**Sex, no. (%)^b^**							
Male	4,511 (47.7)	16,461 (50.0)	<.001	4,475 (48.0)	4,504 (48.3)	.67	0.62
Female	4,939 (52.3)	16,462 (50.0)		4,854 (52.0)	4,825 (51.7)		0.62
**Age, mean (SD), y^b^**	60.7 (11.4)	62.9 (12.1)	<.001	60.8 (11.4)	60.7 (12.3)	.62	0.93
**Age category, no. (%), y**							
<55	3,039 (32.2)	9,152 (27.8)	<.001	2,966 (31.8)	3,229 (34.6)	<.001	5.99
55–64	2,840 (30.1)	8,430 (25.6)		2,806 (30.1)	2,507 (26.9)		7.12
65–74	2,583 (27.3)	9,613 (29.2)		2,572 (27.6)	2,367 (25.4)		4.99
≥75	988 (10.5)	5,728 (17.4)		985 (10.6)	1,226 (13.1)		7.99
**Highest level of education, no. (%)^b^**							
Illiterate	1,276 (13.5)	5,396 (16.4)	<.001	1,268 (13.6)	1,282 (13.7)	.86	0.44
Elementary school	4,515 (47.8)	15,540 (47.2)		4,465 (47.9)	4,437 (47.6)		0.60
High school	2,797 (29.6)	9,133 (27.7)		2,747 (29.5)	2,785 (29.9)		0.88
College or university and above	862 (9.2)	2,854 (8.7)		849 (9.1)	825 (8.8)		0.91
**Residence, no. (%)^b^**							
Urban area	4,394 (46.5)	16,264 (49.4)	<.001	4,354 (46.7)	4,401 (47.2)	.75	1.02
Suburban area	3,976 (42.1)	12,875 (39.1)		3,910 (41.9)	3,886 (41.7)		0.51
Rural area	1,080 (11.4)	3,784 (11.5)		1,065 (11.4)	1,042 (11.2)		0.79
**Baseline comorbidity**							
DCSI, mean (SD)^b^	1.16 (1.34)	1.15 (1.46)	.84	1.16 (1.34)	1.15 (1.45)	.72	0.72
DCSI category, no. (%)							
0	3,852 (40.8)	14,928 (45.3)	<.001	3,803 (40.8)	4,188 (44.9)	<.001	8.33
1	2,666 (28.2)	7,800 (23.7)		2,631 (28.2)	2,273 (24.4)		8.73
≥2	2,932 (31.0)	10,195 (31.0)		2,895 (31.0)	2,868 (30.7)		0.63
**CIC, mean (SD)^b^**	1.10 (0.95)	1.11 (0.98)	.08	1.09 (0.95)	1.09 (0.98)	.99	0
**CIC category, no. (%)**							
0	2,903 (30.7)	10,258 (31.2)	.13	2,871 (30.8)	2,972 (31.9)	.25	2.33
1	3,567 (37.8)	12,058 (36.6)		3,519 (37.7)	3,437 (36.8)		1.82
≥2	2,980 (31.5)	10,607 (32.2)		2,939 (31.5)	2,920 (31.3)		0.43
**Antidiabetes Drug Use at Baseline, No. (%)^c^**							
**Metformin^b^**							
No	2,217 (23.5)	11,127 (33.8)	<.001	2,216 (23.8)	2,191 (23.5)	.67	0.61
Yes	7,233 (76.5)	21,796 (66.2)		7,113 (76.3)	7,138 (76.5)		0.61
**Sulfonylureas^b^**							
No	1,590 (16.8)	6,373 (19.4)	<.001	1,581 (17.0)	1,601 (17.2)	.70	0.56
Yes	7,860 (83.2)	26,550 (80.6)		7,748 (83.1)	7,728 (82.8)		0.56
**Thiazolidinediones^b^**							
No	7,497 (79.3)	28,604 (86.9)	<.001	7,453 (79.9)	7,500 (80.4)	.39	1.25
Yes	1,953 (20.7)	4,319 (13.1)		1,876 (20.1)	1,829 (19.6)		1.25
**α Glucosidase inhibitors^b^**							
No	7,982 (84.5)	29,774 (90.4)	<.001	7,928 (85.0)	7,929 (85.0)	.98	0.03
Yes	1,468 (15.5)	3,149 (9.6)		1,401 (15.0)	1,400 (15.0)		0.03
**Antidiabetes Drug Use During Follow-up, No. (%)^d^**							
**Metformin**							
No	708 (7.5)	4,764 (14.5)	<.001	705 (7.6)	1,092 (11.7)	<.001	NA
Yes	8,742 (92.5)	28,159 (85.5)		8,624 (92.4)	8,237 (88.3)		NA
**Sulfonylureas**							
No	854 (9.0)	3,740 (11.4)	<.001	847 (9.1)	972 (10.4)	.002	NA
Yes	8,596 (91.0)	29,183 (88.6)		8,482 (90.9)	8,357 (89.6)		NA
**Thiazolidinediones**							
No	4,541 (48.1)	20,708 (62.9)	<.001	4,515 (48.4)	5,279 (56.6)	<.001	NA
Yes	4,909 (52.0)	12,215 (37.1)		4,814 (51.6)	4,050 (43.4)		NA
**α Glucosidase inhibitors**							
No	5,035 (53.3)	21,126 (64.2)	<.001	4,998 (53.6)	5,637 (60.4)	<.001	NA
Yes	4,415 (46.7)	11,797 (35.8)		4,331 (46.4)	3,692 (39.6)		NA
**Health Care Facility Characteristics**							
**Accreditation level, no. (%)^b^**							
Medical center	1,915 (20.3)	8,333 (25.3)	<.001	1,915 (20.5)	1,762 (18.9)	.03	4.12
Regional hospital	3,689 (39.0)	8,132 (24.7)		3,584 (38.4)	3,697 (39.6)		2.48
Local hospital	2,172 (23.0)	6,694 (20.3)		2,156 (23.1)	2,140 (22.9)		0.40
Clinic	1,674 (17.7)	9,764 (29.7)		1,674 (17.9)	1,730 (18.5)		1.55
**Geographic region, no. (%)^b^^,^ e**							
Taipei	2,535 (26.8)	10,068 (30.6)	<.001	2,535 (27.2)	2,584 (27.7)	.65	1.19
Northern	1,263 (13.4)	4,838 (14.7)		1,263 (13.5)	1,304 (14.0)		1.28
Central	3,296 (34.9)	5,361 (16.3)		3,175 (34.0)	3,107 (33.3)		1.54
Southern	852 (9.0)	4,955 (15.1)		852 (9.1)	839 (9.0)		0.49
Kao-Ping	1,269 (13.4)	6,732 (20.5)		1,269 (13.6)	1,284 (13.8)		0.47
Eastern	235 (2.5)	969 (2.9)		235 (2.5)	211 (2.3)		1.70

Abbreviations: CIC, chronic illness with complexity index (20); DCSI, diabetes complications severity index (19); NA, not applicable; P4P, pay-for-performance program; PSM, propensity score matching.

a Standardized difference was used to compare the balancing for the mean or frequencies of a covariate that were included in the propensity score matching process (18). Standardized difference was calculated to evaluate the efficiency of PSM.

b These variables were used in the PSM.

c Baseline antidiabetes drug use was measured if patients were prescribed the selected medications within 1 year before index date; patients could use more than 1 drug.

d Follow-up antidiabetes drug use was measured if patients were prescribed the selected medications between the index date and the study end date (December 31, 2010), cancer occurrence, or death date, whichever came first; patients could use more than 1 drug. This set of variables was not included in the PSM regression.

e Geographic region categories were based on the branches of the National Health Insurance Administration in which the hospitals were located.

## Results

Before PSM 1-to-1 matching, the P4P group and the non-P4P group differed significantly (*P* < .001) by several characteristics (Table 1). After matching, all baseline characteristics between the 2 matched groups were similar. Of the P4P patients, 48.0% were men and had a mean age of 60.8 years; 38.6% had an education level of high school or above, and 46.7% lived in urban areas. Mean DCSI was 1.16 and mean was CIC 1.09. Baseline antidiabetes drug use did not differ significantly between P4P and non-P4P patients. Compared with non-P4P patients, P4P patients received more metformin, TZDs, and AGIs during follow-up (*P* < .001).

Table 2 compares total person-years and crude cancer incidence and mortality per 1,000 person-years for various end points between P4P and non-P4P patients. Risks of overall cancer incidence were not significantly associated with the P4P program but were associated with use of antidiabetes medication, particularly metformin (aSHR, 0.58: 95% CI, 0.50–0.67), TZDs (aSHR, 0.78; 95% CI, 0.70–0.86), and AGIs (aSHR, 0.62; 95% CI, 0.55–0.69) (Table 3). P4P patients had lower risks of all-cause mortality (aSHR, 0.59; 95% CI, 0.55–0.63), cancer-specific mortality (aSHR, 0.85; 95% CI, 0.73–1.00), and diabetes-related mortality (aSHR, 0.54; 95% CI, 0.49–0.60) than non-P4P patients with diabetes (Figure).

**Table 2:** Incidence of Cancer, Mortality, and Total Person-Years for Matched P4P and Non-P4P Patients With Type 2 Diabetes, Taiwan

Outcome	Total No. Person-Years Studied		Events			Incidence Per 1,000 Person-Years Studied		IRR^a^	
	P4P	Non-P4P	P4P, N (%)	Non-P4P, N (%)	*P* Value	P4P	Non-P4P	IRR (95% CI)	*P* Value
**No.**	9,329	9,329	9,329	9,329	NA	NA			
**Cancer incidence**									
Overall	59,564	56,790	800 (8.6)	826 (8.9)	.50	13.43	14.54	0.92 (0.84–1.02)	.11
Breast	61,144	58,203	54 (1.1)	62 (1.3)	.44	0.88	1.07	0.83 (0.56–1.21)	.32
Colorectal	61,050	58,148	112 (1.2)	113 (1.2)	.95	1.83	1.94	0.94 (0.72–1.24)	.67
Oral	61,237	58,302	34 (0.4)	35 (0.4)	.90	0.56	0.60	0.93 (0.56–1.53)	.75
Liver	61,059	58,124	157 (1.7)	191 (2.1)	.07	2.57	3.29	0.78 (0.63–0.97)	.02
Lung	61,216	58,266	76 (0.8)	88 (0.9)	.35	1.24	1.51	0.82 (0.60–1.13)	.21
Cervical	61,231	58,307	29 (0.3)	28 (0.3)	.90	0.47	0.48	0.99 (0.57–1.72)	.96
Prostate	61,227	58,298	35 (0.4)	34 (0.4)	.90	0.57	0.58	0.98 (0.59–1.62)	.93
Stomach	61,254	58,323	44 (0.5)	43 (0.5)	.91	0.72	0.74	0.97 (0.63–1.52)	.90
Bladder	61,230	58,317	29 (0.3)	35 (0.4)	.45	0.47	0.60	0.79 (0.47–1.33)	.35
**Mortality**									
All-cause mortality	61,324	58,389	1,258 (13.5)	2,099 (22.5)	<.001	20.51	35.95	0.57 (0.53–0.61)	<.001
Cancer-specific mortality	61,324	58,389	283 (3.0)	347 (3.7)	.010	4.61	5.94	0.78 (0.66–0.91)	.002
Diabetes-related mortality	61,324	58,389	541 (5.8)	988 (10.6)	<.001	8.82	16.92	0.52 (0.47–0.58)	<.001

Abbreviations: IRR, incidence rate ratio; NA, not applicable; P4P, pay-for-performance program.

a The non-P4P group is the reference group.

**Table 3:** Adjusted Subdistribution Hazard Ratios (aSHRs) for the Effects of a P4P Program and Antidiabetes Medications on the Risks of Cancer Incidence, Taiwan^a^

Cancer	Program Effects, P4P^c^		Antidiabetes Medication Effects^b^							
			Metformin		SUs		TZDs		AGIs	
	aSHR (95% CI)	*P* Value	aSHR (95% CI)	*P* Value	aSHR (95% CI)	*P* Value	aSHR (95% CI)	*P* Value	aSHR (95% CI)	*P* Value
Overall	1.04 (0.94–1.15)	.42	0.58 (0.50–0.67)	<.001	1.05 (0.90–1.23)	.53	0.78 (0.70–0.86)	<.001	0.62 (0.55–0.69)	<.001
Breast	0.93 (0.64–1.35)	.71	0.81 (0.46–1.40)	.44	0.67 (0.40–1.13)	.13	0.76 (0.50–1.14)	.18	0.60 (0.40–0.91)	.02
Colorectal	1.09 (0.83–1.42)	.53	0.52 (0.36–0.74)	<.001	1.81 (1.12–2.93)	.02	0.58 (0.43–0.79)	.001	0.60 (0.44–0.81)	.001
Oral	1.08 (0.65–1.78)	.78	0.61 (0.34–1.09)	.10	0.89 (0.47–1.69)	.72	0.83 (0.49–1.41)	.50	0.64 (0.39–1.06)	.08
Liver	0.91 (0.73–1.12)	.37	0.48 (0.36–0.63)	<.001	1.11 (0.80–1.53)	.54	0.76 (0.60–0.96)	.02	0.47 (0.36–0.61)	<.001
Lung	0.91 (0.67–1.24)	.56	0.91 (0.56–1.49)	.72	1.57 (0.90–2.77)	.12	0.84 (0.60–1.18)	.31	0.60 (0.42–0.86)	.005
Cervical	1.15 (0.68–1.97)	.60	0.44 (0.20–0.94)	.03	0.79 (0.34–1.88)	.60	0.75 (0.43–1.31)	.31	0.72 (0.42–1.24)	.24
Prostate	1.06 (0.67–1.70)	.80	0.69 (0.37–1.29)	.24	1.34 (0.64–2.80)	.43	0.67 (0.39–1.15)	.15	0.38 (0.20–0.73)	.004
Stomach	1.10 (0.72–1.67)	.66	0.59 (0.33–1.06)	.08	1.04 (0.53–2.07)	.90	1.02 (0.66–1.60)	.92	0.78 (0.48–1.25)	.30
Bladder	0.82 (0.49–1.36)	.44	0.73 (0.34–1.60)	.43	1.20 (0.49–2.93)	.69	0.75 (0.43–1.29)	.30	0.59 (0.33–1.03)	.07

Abbreviations: AGIs, α glucosidase inhibitors; CI, confidence interval; P4P, pay-for-performance program; SUs, sulfonylureas; TZDs, thiazolidinediones.

a Competing risk regression models were used to analyze the effects of P4P and drug effects on risks of cancer incidences while controlling for potential confounders: age, sex, highest level of education, rural or urban residence, baseline comorbidity (diabetes complications severity index and chronic illness with complexity), antidiabetes drug use (metformin, SUs, TZDs, AGIs), and health care facility characteristics (accreditation level and geographic regions).

b Nonusers of the drug are the reference group for each drug.

c The non-P4P group is the reference group.

**Figure:**
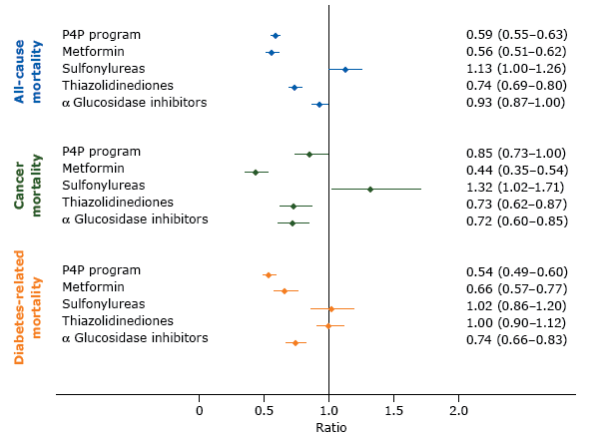
Adjusted model results and 95% confidence intervals for the effects of a pay-for-performance program (P4P) and prescribed antidiabetes medications on the ­risks of all-cause, cancer, and diabetes-related mortality in Taiwan. Competing risk regression models were used to analyze the effects of P4P and drug effects on risks of cancer-specific and diabetes-related mortality and the adjusted subdistribution hazard ratios were calculated. A Cox proportionate hazard model was used to analyze all-cause mortality and the adjusted hazard ratios were calculated. Potential confounders that were controlled for were age, sex, highest level of education, rural or urban residence, baseline comorbidity (diabetes complications severity index and chronic illness with complexity), antidiabetes drug use (metformin, sulfonylureas, thiazolidinediones, α glucosidase inhibitors), and health care facility characteristics (accreditation level and geographic regions). **Table:** 

Effect	Adjusted Subdistribution Hazard Ratio (95% Confidence Interval)
**All-cause mortality**	
P4P program	0.59 (0.55–0.63)
Metformin	0.56 (0.51–0.62)
Sulfonylureas	1.13 (1.00–1.26)
Thiazolidinediones	0.74 (0.69–0.80)
α Glucosidase inhibitors	0.93 (0.87–1.00)
**Cancer mortality**	
P4P program	0.85 (0.73–1.00)
Metformin	0.44 (0.35–0.54)
Sulfonylureas	1.32 (1.02–1.71)
Thiazolidinediones	0.73 (0.62–0.87)
α Glucosidase inhibitors	0.72 (0.60–0.85)
**Diabetes-related mortality**	
P4P program	0.54 (0.49–0.60)
Metformin	0.66 (0.57–0.77)
Sulfonylureas	1.02 (0.86–1.20)
Thiazolidinediones	1.00 (0.90–1.12)
α Glucosidase inhibitors	0.74 (0.66–0.83)

## Discussion

Recent literature has focused on cancer risk or mortality with various diabetes treatments, given the potential biologic link or common risk factors for the observed associations between type 2 diabetes and cancer (3–6). However, studies on the effect of diabetes control through a comprehensive diabetes P4P program on cancer risks or mortality were inconclusive (4–6,24). Overall, our findings indicated that the diabetes P4P program was not significantly associated with lower risks of cancer incidence, but it was associated with lower risks of all-cause mortality, cancer-specific mortality, and diabetes-related mortality.

With all other variables constant, our findings suggest that the diabetes P4P program may be less likely to affect the risk of overall or specific cancer incidence while several types of glucose-lowering therapies (metformin, TZDs, AGIs) may have a protective effect, consistent with results of observational studies (25,26). The nonsignificant association between the P4P program and cancer incidence might be explained by several reasons. First, compared with medications that affect the potential biologic link between diabetes and cancer risk within a shorter period, medications used over a longer follow-up period may be required for a comprehensive disease management program to demonstrate reduction in risk. Second, previous studies found that, after enrollment, P4P patients had significantly more outpatient visits, greater expense, and higher usage of guideline-recommended services (including preventive services) than non-P4P patients (21). Thus, P4P patients may receive more cancer screening tests and have more early cancer detection and treatment. The potential effect of a comprehensive disease management program on cancer risk might be mitigated. Future study is suggested to examine the effect of a diabetes P4P program on incidence of specific cancers.

Alternatively, our findings suggest that the diabetes P4P program not only has a protective effect on all-cause mortality and diabetes-related mortality but also influences cancer-specific mortality among incident cancer patients. Cancer survival outcomes among P4P patients might be explained by several reasons. First, the diabetes P4P program in Taiwan requires participating health care providers to adhere to the American Diabetes Association’s clinical practice guidelines. The guidelines are consistent with the nutrition and physical activity guidelines for cancer survivors suggested by the American Cancer Society, including recommendations for psychosocial and social assessment and care, preventive care (vaccinations, screening), lifestyle changes, diet, physical activities, and obesity management (27). As studies by Chiao et al (28) and Calip et al (29) suggested, well diabetes care and glucose control before cancer diagnosis (colorectal, breast), which persisted after diagnosis, may have moderated the mortality effect of diabetes in newly diagnosed cancer patients (28,29). Second, in contrast to the uncertain relationship between glucose-lowering treatments and cancer incidence, previous studies supported beneficial effects of antidiabetes medications, along with glycemic control, possibly affecting cancer treatments, which may in turn influence cancer-specific mortality (4,25,29). We observed that P4P patients tended to have more opportunity to receive oral glucose-lowering medications (metformin, TZDs, AGIs) than non-P4P patients during follow-up (all *P* < .001; Table1). Consistent with other studies, our study (Figure) indicated that metformin (aSHR, 0.44: 95% CI, 0.35–0.54), TZDs (aSHR, 0.73; 95% CI, 0.62–0.87), and AGIs (aSHR, 0.72; 95% CI, 0.60–0.85) were associated with lower risks of cancer-specific mortality in incident cancer patients.

This study has several limitations. First, given the limitation of our study data periods in the administrative claims from 2000, we could not track the number of years since diabetes was diagnosed or identify whether patients were newly diagnosed. However, we could track the time of cancer diagnosis through the cancer registry data set since 1979 as the washout period for exclusion criteria. Second, some unobservable confounders for individual patients (eg, lifestyle, prescription adherence, illness experience, smoking status, body weight, psychological and social assessment) were unavailable in the secondary database. Although these baseline confounding factors were not part of the study design and we assumed that all characteristics related to health outcomes in the P4P program were covered by the measured variables among P4P and non-P4P patients, caution is necessary when interpreting the effect of the P4P program on mortality. Third, given our study design, we observed cancer incidence and mortality events over a 5-year period. A greater effect might have been found with a longer observation period. Nevertheless, our 5-year observation period was considered sufficient to evaluate and compare various quality improvement interventions (30). Finally, the data we used were obtained from patients with diabetes in Taiwan, so results may not be generalized to other P4P programs in other countries.

Despite these limitations, this study addressed the issue of whether integrated interventions through a comprehensive and multidisciplinary diabetes P4P program might mitigate cancer risks and mortality. Compared with patients with diabetes not enrolled in the P4P program, P4P patients had lower risk of all-cause mortality, cancer-specific mortality, and diabetes-related mortality, but the groups did not differ in overall risks of incident cancer. Our findings provide evidence of the potential benefit of diabetes P4P programs in reducing risks of all-cause mortality and competing causes of death attributable to cancer-specific and diabetes-related mortality among type 2 diabetes patients.
